# Electroacupuncture preconditioning reduces cerebral ischemic injury via BDNF and SDF-1α in mice

**DOI:** 10.1186/1472-6882-13-22

**Published:** 2013-01-28

**Authors:** Ji Hyun Kim, Kyung Ha Choi, Young Jung Jang, Ha Neui Kim, Sun Sik Bae, Byung Tae Choi, Hwa Kyoung Shin

**Affiliations:** 1 Division of Meridian and Structural Medicine, School of Korean Medicine, Pusan National University, Yangsan, Gyeongnam, 626-870, Republic of Korea; 2 Department of Pharmacology, School of Medicine, Pusan National University, Yangsan, Gyeongnam, 626-870, Republic of Korea

**Keywords:** Focal cerebral ischemia, Electroacupuncture, BDNF, SDF-1α, Preconditioning

## Abstract

**Background:**

This study was designed to determine if electroacupuncture (EA) preconditioning improves tissue outcome and functional outcome following experimentally induced cerebral ischemia in mice. In addition, we investigated whether the expression of brain-derived neurotrophic factor (BDNF) and stromal cell derived factor-1α (SDF-1α) and infarct volume were related with improvement in neurological and motor function by interventions in this study.

**Methods:**

After treatment with EA at the acupoints ‘Baihui (GV20)’ and ‘Dazhui (GV14)’ for 20 min, BDNF was assessed in the cortical tissues based on Western blot and the SDF-1α and vascular endothelial growth factor (VEGF) levels in the plasma determined by ELISA. To assess the protective effects of EA against ischemic injury, the mice received once a day 20 min EA preconditioning for three days prior to the ischemic event. Focal cerebral ischemia was then induced by photothrombotic cortical ischemia. Infarct volumes, neurobehavioral deficit and motor deficit were evaluated 24 h after focal cerebral ischemia.

**Results:**

The expression of BDNF protein increased significantly from 6 h, reaching a plateau at 12 h after the end of EA treatment in the cerebral cortex. Furthermore, SDF-1α, not VEGF, increased singnificantly from 12 h to 48 h after EA stimulation in the plasma. Moreover, EA preconditioning reduced the infarct volume by 43.5% when compared to control mice at 24 h after photothrombotic cortical ischemia. Consistent with a smaller infarct size, EA preconditioning showed prominent improvement of neurological function and motor function such as vestibule-motor function, sensori-motor function and asymmetric forelimb use. The expression of BDNF colocalized within neurons and SDF-1α colocalized within the cerebral vascular endothelium was observed throughout the ischemic cortex by EA.

**Conclusions:**

Pretreatment with EA increased the production of BDNF and SDF-1α, which elicited protective effects against focal cerebral ischemia. These results suggest a novel mechanism of EA pretreatment-induced tolerance against cerebral ischemic injury.

## Background

Despite decades of intense research, the treatment of strokes by anticoagulation, thrombolysis and neuroprotection have not been found to fully improve stroke patients. Electroacupuncture (EA), a traditional therapy, has been recommended as a complementary therapy for pain relief [1] and stroke rehabilitation [2]. Several studies investigating the effectiveness of EA with cerebral ischemia have been conducted and beneficial outcomes were observed in experimental animals [3-8]. Therefore, EA presumably improves the outcome of stroke patients; however, the cellular mechanisms underlying this improvement remain elusive.

In the pretreatment effect, a brief exposure to sublethal or noninjurious stimuli increases resistance to subsequent prolonged and lethal damage [9]. EA pretreatment has recently been shown to induce ischemic tolerance in a fashion mimicking the ischemia pretreatment [10,11]. Since EA is economical, easily conducted, and has fewer negative side effects than other prevention methods (e.g., pharmacological, ischemic, etc.), it should be valuable and advantageous to preventing ischemic cerebrovascular disease, especially in patients at high risk of ischemic injury. Many studies have shown that protective mechanisms of EA pretreatment may involve a series of regulatory molecular pathways including enhanced antioxidant activity [8], regulation of the endocannabinoid system [12], and involvement of the postreceptor signaling pathway [13]. However, the mechanism is not fully understood and more evidence is needed for pretreatment with EA to be accepted clinically.

Neurotrophic factors not only contribute to protection in the acute phase and neurogenesis in the chronic phase after cerebral ischemia, but also provide preconditioning-induced ischemic tolerance [14]. Among neurotrophic factors, brain-derived neurotrophic factor (BDNF) and stromal cell derived factor-1α (SDF-1α) are thought to be potent candidates in the recovery from cerebral ischemia [15-17]. However, little is known about the involvement of BDNF and SDF-1α in EA preconditioning in ischemic brains.

Therefore, the present study was conducted to investigate whether EA preconditioning at the Baihui (GV20) and Dazhui (GV14) acupoints improves tissue outcome and functional outcome following experimentally induced cerebral ischemia in mice. Furthermore, we investigated whether the expression of BDNF and SDF-1α and infarct volume were related to the functional recovery by interventions.

## Methods

### General surgical preparation

Male mice (C57BL/6J, 20–25 g) were housed under diurnal lighting conditions and allowed food and tap water *ad libitum*. This study was carried out in strict accordance with the recommendations in the Guide for the Care and Use of Laboratory Animals of the National Institutes of Health. The protocol was approved by the Pusan National University Institutional Animal Care and Use Committee (Permit Number: PNU-2011-000420). Anesthesia was achieved by face mask-delivered isoflurane (2% induction and 1.5% maintenance, in 80% N_2_O and 20% O_2_).

### Electroacupuncture stimulation

**Figure 1:**
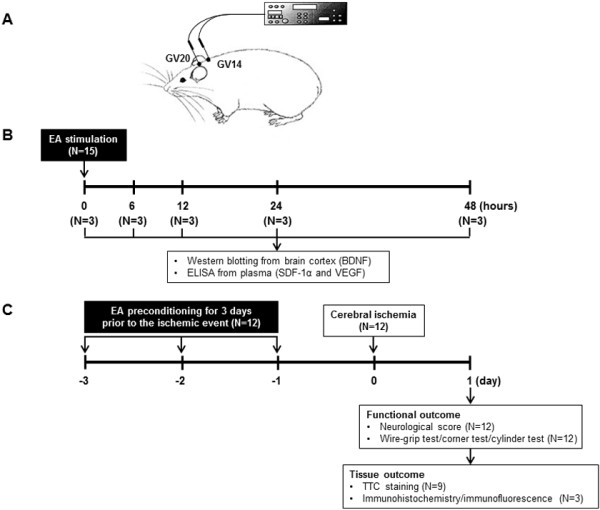
**Summary of experimental protocols. (A)** Mouse schematic showing the location of the acupuncture points used in the study. GV20 stands for ‘Baihui’, which is located at the right midpoint of the parietal bone, GV14 stands for ‘Dazhui’, which is located on the posterior midline and in the depression below the spinous process of the seventh cervical vertebra. **(B)** The time line showing the chronological events of western blotting and ELISA studies. The brain tissue and blood were collected at 6, 12, 24 and 48 h after the end of EA treatment. **(C)** The time line showing the chronological events of the protective effects of EA on ischemic injury. The mice received once a day 20 min EA preconditioning for three days prior to the ischemic event, then focal cerebral ischemia was induced by photothrombotic cortical ischemia. Infarct volumes, neurobehavioral deficit and motor deficit were evaluated 24 h after focal cerebral ischemia.

Animals were anesthetized with isoflurane to avoid restraint stress. The transpositional method was used to determine the acupoints in mice, which locates the veterinary acupoints by transforming human acupoints onto animal anatomy [18]. The acupoint ‘Baihui (GV20)’, which is located at the right midpoint of the parietal bone, and ‘Dazhui (GV14)’, which is located on the posterior midline and in the depression below the spinous process of the seventh cervical vertebra, were stimulated as standard criteria in mouse (Figure 1A). To accomplish this acupuncture needles (0.18 × 30 mm) were inserted into the GV20 and GV14 to the depth of approximately 3 mm, after which the acupoints were stimulated at an intensity of 1 mA and a frequency of 2 Hz for 20 min using Grass S88 electro stimulator (Grass Instrument Co., West Warwick, RI). The intensity was maintained just below the level that induced visible muscle contraction. The control groups received the same electrical stimulation at non-acupuncture points. To assess the protective effects of EA on ischemic injury, the mice received once a day 20 min EA preconditioning for three days prior to the ischemic event (Figure 1C).

### Western blotting

Each mouse was deeply anesthetized with thiopental sodium at 6, 12, 24 and 48 h after EA stimulation (Figure 1B), subsequently perfused transcardially with cold PBS and brain cortex was collected. Immunoblot analysis was performed with BDNF antibodies (Millipore, Temecula, CA) followed by incubation with secondary antibody conjugated with horseradish peroxidase. The intensity of chemiluminescence was measured using an ImageQuant LAS 4000 apparatus (GE Healthcare Life Sciences, Uppsala, Sweden).

### Measurement of SDF-1α and VEGF in the plasma

To determine the effects of EA treatment on SDF-1α and vascular endothelial growth factor (VEGF) production, blood sample was collected through cardiac puncture at 6, 12, 24 and 48 h after the end of EA treatment (Figure 1B). Blood sample was taken from the heart, preferably from the ventricle slowly to avoid collapsing of heart. The plasma was separated and kept at −80°C until assay. The amount of SDF-1α and VEGF from plasma was determined using a commercially available ELISA kit according to the manufacturer’s instructions (R&D Systems, Minneapolis, MN). The color intensity was determined using a SpectraMax190 microplate reader (Molecular Devices, Sunnyvale, CA).

### Focal cerebral ischemia

Focal cerebral ischemia was induced by a photothrombotic cortical ischemia model [19]. Rose Bengal (Sigma-Aldrich; 0.1 ml of a 10 mg/ml solution in sterile saline) was injected intraperitoneally 5 min before illumination. After mice were placed in a stereotaxic frame, a midline scalp was incised, pericranial tissues were dissected, and the bregma and lambda points were identified. A fiber optic bundle of a KL1500 LCD cold light source (Carl Zeiss, Jena, Germany) with a 4 mm aperture was centered using a micromanipulator 2 mm laterally from the bregma. The brain was illuminated through the intact skull for 15 min, after which the surgical wound was sutured and mice were allowed to recover from anesthesia. The brains were removed 24 h after ischemia insults. The cerebral infarct size was then determined on 2,3,5-triphenyltetrazolium chloride (TTC)-stained, 2-mm-thick brain sections. Infarction areas were quantified with iSolution full image analysis software (Image & Microscope Technology, Vancouver, Canada). To account for and eliminate the effects of swelling/edema, the infarction volume was calculated using an indirect measurement in which the volumes of each section were summed according to the following formula: contralateral hemisphere (mm^3^) - undamaged ipsilateral hemisphere (mm^3^).

### Neurological score

Neurological deficit was scored in each mouse at 24 h after the ischemic insult in a blinded fashion according to the following graded scoring system: 0 = no deficit; 1 = forelimb weakness and torso turning to the ipsilateral side when held by tail; 2 = circling to the affected side; 3 = unable to bear weight on the affected side; and 4 = no spontaneous locomotor activity or barrel rolling [20].

### Wire-grip test

Vestibulo-motor function was assessed using a wire-grip test 24 h after cerebral ischemia [21]. Briefly, mice were placed on a metal wire (45 cm long) suspended 45 cm above protective padding and allowed to traverse the wire for 60 sec. The latency for which a mouse remained on the wire within a 60-sec interval was measured, and the wire grip score was quantified using the 5-point scale.

### Corner test

This test detects integrated sensori-motor function as it involves both stimulation of the vibrissae (sensory/neglect) and rearing (motor response) as previously described [22]. The mouse was placed between two cardboard pieces, each with a dimension of 30 × 20 × 1 cm^3^. The two boards were gradually moved closer to the mouse from both sides to encourage it to enter into a corner of 30° with a small opening along the joint between the two boards. When the mouse entered into the deep the part of the corner, both sides of the vibrissae were stimulated together by the two boards. The mouse then reared forward and upward, after which it turned back to face the open end. Twenty trials were performed for each mouse and the percentage of right turns was calculated.

### Cylinder test

The cylinder test was adapted for use in mice to assess forelimb use and rotation asymmetry [23]. Briefly, the mouse was placed in a transparent cylinder 9-cm in diameter and 15 cm tall. After the mouse was put into the cylinder, forelimb use of the first contact against the wall after rearing and during lateral exploration was recorded. The final score = (nonimpaired forelimb movement - impaired forelimb movement)/(nonimpaired forelimb movement + impaired forelimb movement + both movement). This test evaluates forelimb use asymmetry for weight shifting during vertical exploration and provides high inter-rater reliability with even inexperienced raters.

### Immunohistochemistry and immunofluorescence

Twenty four hours after cerebral ischemia, mice deeply anesthetized with thiopental sodium were perfused transcardially with cold PBS followed by 4% paraformaldehyde for fixation. The brain of each mouse was then removed and further fixed for 24 h in 4% paraformaldehyde at 4°C followed by cryoprotection in 20% sucrose for 72 h at 4°C. The frozen brains were cut at a thickness of 14 μm using a Leica CM 3050 cryostat (Leica Microsystes, Wetzlar, Germany). For immunohistochemistry, the sections were stained with antibody against BDNF (Millipore) or SDF-1α (R&D Systems). Following additional incubation with biotinylated secondary antibody, samples were incubated in ABC reagent (Vector Laboratories, Burlingame, CA). All reactions were visualized by development in 3,3’ diaminobenzidine substrates (Vector Laboratories), and all samples were visualized using a light microscope (Carl Zeiss, Jena, Germany). The immunoreaction products were quantified using the iSolution full image analysis software. In double-fluorescence staining, sections were stained with anti-NeuN (Chemicon, Temecula, CA) or anti-CD31 antibody (BD Biosciences, Franklin Lakes, NJ), after which they were treated with FITC-conjugated secondary antibody to detect NeuN or CD31, and subsequently with anti-BDNF or SDF-1α antibody, followed by treatment with Texas red-conjugated secondary antibody to detect BDNF or SDF-1α. Fluorescent-stained sections were analyzed by Axio Imager fluorescence microscopy (Carl Zeiss, Jena, Germany).

### Data analysis

The data are expressed as mean ± standard error of mean (SEM). BDNF, SDF-1α and VEGF levels after EA stimulation was analyzed by one-way analysis of variance (ANOVA) and control vs. EA group was compared by unpaired *t*-test. The differences were considered statistically significant, when the two-tailed p values were less than 0.05. Statistical analysis was performed using SigmaPlot 11.2 (Systat Software Inc, San Jose, CA).

## Results

### Effect of EA on BDNF, SDF-1α and VEGF production

**Figure 2:**
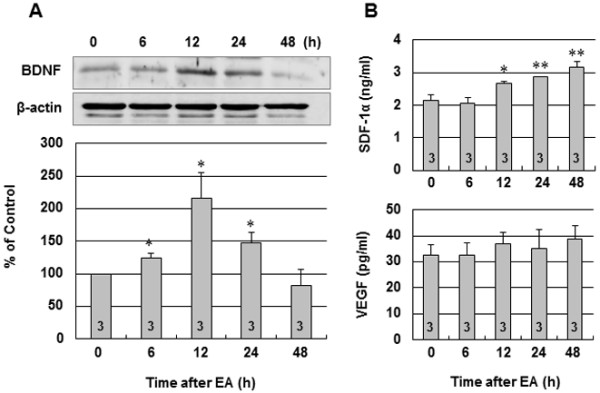
**Temporal production of BDNF in the brain tissues and SDF-1α and VEGF levels in the plasma following EA. (A)** Representative western blot of BDNF protein in the cerebral cortex at 0 h (control), 6 h, 12 h, 24 h, and 48 h after the end of EA stimulation. The lower panel shows densitometric analysis of the western blot of BDNF. Relative abundance of BDNF compared with β-actin. **(B)** Plasma SDF-1α and VEGF levels in mice at 0 h (control), 6 h, 12 h, 24 h, and 48 h after the end of EA stimulation were analyzed by ELISA. The results are expressed as the mean ± SEM. *, *P*<0.05 and **, *P*<0.01 vs. control. Numbers of mice used for BDNF, SDF-1α and VEGF measurement are shown on each bar.

Using a time course of EA treatment, BDNF was assessed in the brain tissues from identical cortical areas by Western blot. The expression of BDNF protein significantly increased from 6 h, reaching a plateau at 12 h after the end of EA treatment (216.32±38.97%, *P*<0.05 vs. control). After 24 h, BDNF protein levels began to decrease, returning to near the control level by 48 h (Figure 2A). Figure 2B illustrates the time course of SDF-1α and VEGF levels in the plasma at 6, 12, 24 and 48 h after EA stimulation. Treatment with EA gradually increased the plasma content of SDF-1α from 12 h to 48 h (3.18±0.15 ng/ml vs. 2.15±0.17 ng/ml, 48 h and control respectively, *P*<0.01). However, there was no difference in VEGF production in the plasma at 6, 12, 24 and 48 h after the end of EA treatment (Figure 2B).

### Effect of EA preconditioning on tissue outcome and functional outcome

**Figure 3:**
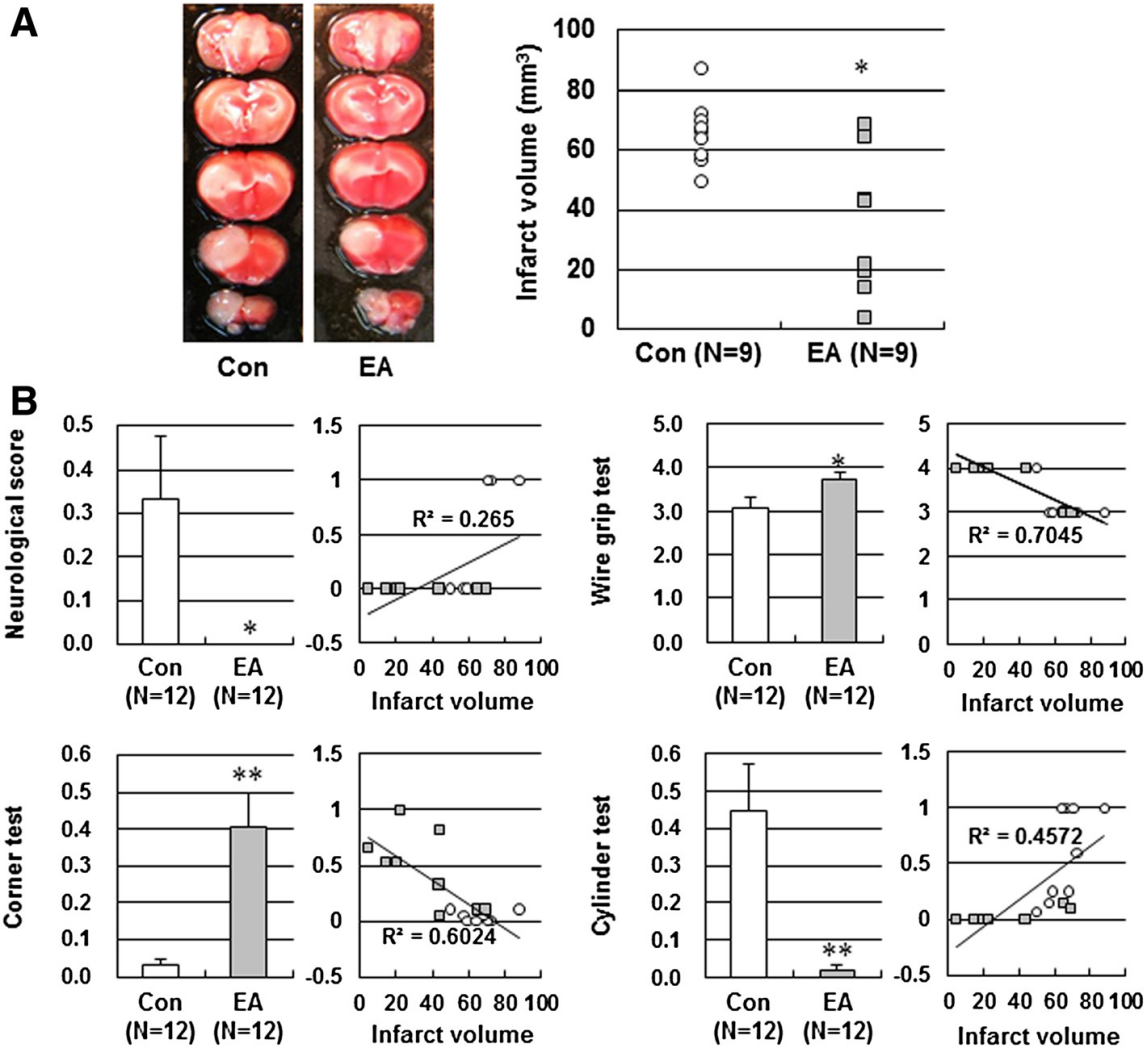
**Effect of EA preconditioning on tissue and functional outcome. (A)** Representative photographs of coronal brain sections following infarction stained with 2,3,5-triphenyltetrazolium chloride. The red area is healthy tissue and the white area is infarct tissue. *, *P*<0.05 vs. control (Con). **(B)** Neurological deficit and motor deficit were assessed 24 h after ischemia. Neurological function was assessed by neurological score, vestibule-motor function by a wire grip test, sensori-motor function by a corner test and asymmetry forelimb use for weight shifting by a cylinder test. A regression line showed the relationship between tissue injury (infarct volume) and functional outcome (neurological score, wire grip test, corner test and cylinder test). Results are expressed as mean ± SEM for twelve mice in each group. *, *P*<0.05 and **, *P*<0.01 vs. control (Con).

To assess the protective effects of EA on ischemic injury, the mice received once a day 20 min EA preconditioning for three days prior to the ischemic event. EA preconditioning reduced the infarct volume by 43.5% when compared to control mice when measured 24 h after photothrombotic cortical ischemia (Figure 3A). Consistent with a smaller infarct size, EA preconditioning showed prominent improvement of neurological function and motor functions (Figure 3B).

### Effect of EA preconditioning on BDNF and SDF-1α expression in ischemic brain

**Figure 4:**
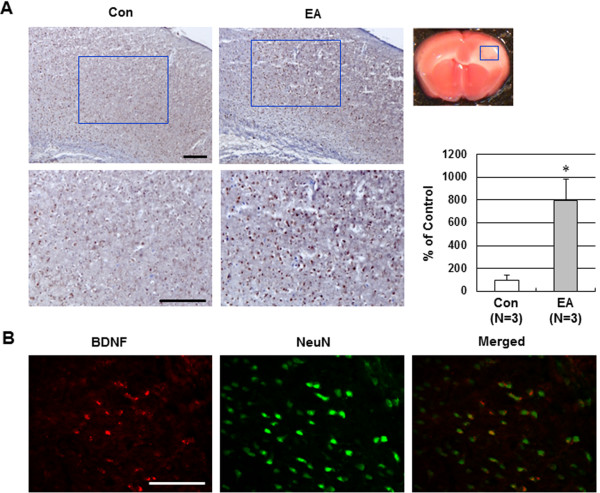
**Effect of EA preconditioning on BDNF expression in ischemic brain. (A)** Representative immunohistochemical staining photographs showed BDNF-positive cell expression 24 h after occlusion in the ischemic cortex of mice that received control or EA. The blue rectangle represents the imaging field. Quantification of BDNF-positive cells is expressed as the % change of the control. The results are expressed as mean ± SEM for three mice in each group. *, *P*<0.05 vs. control (Con). **(B)** Representative double immunofluorescence staining for BDNF (red) and NeuN (neuronal marker, green) in EA-preconditioned ischemic brain. EA-induced BDNF expression was colocalized with the neurons after ischemic injury. Scale bar = 100 μm.

**Figure 5:**
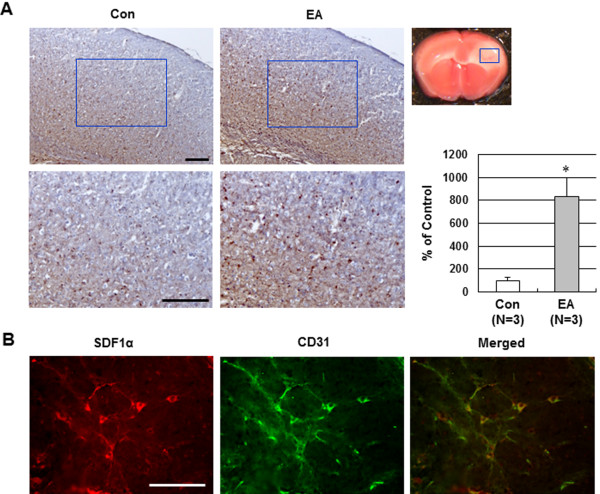
**Effect of EA preconditioning on SDF-1α expression in ischemic brain. (A)** Representative immunohistochemical staining showed SDF-1α-positive cell expression 24 h after occlusion in the ischemic cortex of mice that received control or EA. The blue rectangle represents the imaging field. Quantification of SDF-1α-positive cells is expressed as the % change of the control. The results are expressed as mean ± SEM for three mice in each group. *, *P*<0.05 vs. control (Con). **(B)** Representative double immunofluorescence staining for SDF-1α (red) and CD31 (endothelial cell marker, green) in EA-preconditioned ischemic brain. EA-induced SDF-1α was colocalized with the endothelium after ischemic injury. Scale bar = 100 μm.

To explore the underlying mechanisms of EA preconditioning on focal cerebral ischemia, we investigated BDNF and SDF-1α expression in the ischemic cortex. A large number of BDNF- and SDF-1α-positive cells in the ischemic cortex were observed in the EA group, whereas only a small amount of BDNF- and SDF-1α-positive cells were observed in the control group (Figure 4A and Figure 5A). Double immunofluorescence staining was performed to determine the relationship between BDNF and neuronal cells, or BDNF and endothelial cells. Almost all BDNF-positive cells (red) colocalized with the neuronal cell marker NeuN (green) (Figure 4B), whereas few BDNF-positive cells coexpressing the endothelial phenotype of CD31-positive cells were observed (data not shown). However, double immunofluorescence for SDF-1α and CD31 demonstrated that the SDF-1α was primarily localized in endothelium in the cerebral cortex (Figure 5B).

## Discussion

The present study demonstrated that tolerance to focal cerebral ischemia induced by EA pretreatment is mediated by BDNF and SDF-1α. Our results showed that pretreatment with EA at Baihui (GV20) and Dazhui (GV14) for 20 min increased the brain tissue content of BDNF and upregulated the production of SDF-1α in the plasma. Moreover, EA preconditioning reduced infarct volume by 43.5% when compared to control mice 24 h after photothrombotic cortical ischemia. Consistent with a smaller infarct size, EA preconditioning showed prominent improvement of neurological function and motor function such as vestibule-motor function, sensori-motor function and asymmetry forelimb use. The expression of BDNF, colocalized within neurons, and SDF-1α, colocalized within the cerebral vascular endothelium, was observed throughout the ischemic cortex following EA. These findings provide valuable insight into BDNF and SDF-1α that elicited neuroprotective effects induced by EA preconditioning against focal cerebral ischemia.

Anticoagulation, thrombolysis and cellular protection have not been shown to fully improve stroke patients. EA is a novel therapy based on traditional acupuncture combined with modern electrotherapy. Clinically, acupuncture has shown significant therapeutic benefits for stroke patients [1,2,24,25]. A large number of animal studies have shown that EA could reduce neural apoptosis, promote cell proliferation, increase cerebral blood flow (CBF), and improve neurological function after stroke [4-7]. However, the precise mechanism of EA function remains controversial. In addition, the type of stroke, its severity and the interval after the stroke and pretreatment might also significantly influence the results of EA studies [26].

Preconditioning as a potent endogenous protective response, activates several endogenous signaling pathways that result in tolerance against ischemia [27]. In recent years, numerous studies have shown that EA also have pretreatment effects, including ischemic tolerance as well [10,11]. Since EA is economical, easily performed, and has few negative side effects, it is clinically applicable for prevention, and not just treatment of ischemic cerebral disease. Our study demonstrated that, similar to the ischemic tolerance induced by ischemic preconditioning, repeated EA pretreatment at Baihui (GV20) and Dazhui (GV14) for 20 min a day for three days before lethal ischemic insult could reduce infarct volumes and improve neurological function and motor function 24 h after occlusion. These findings indicate that EA pretreatment could induce tolerance to cerebral ischemic insult.

EA has been reported to increase neurotrophic factors such as insulin-like growth factor 1, basic fibroblast growth factor, glial derived neurotrophic factor or receptors, NMDA NR1 and TRPM7 [13,28-30]. BDNF, SDF-1α and VEGF are hypoxia-inducible factor (HIF)-1 target proteins, which have been implicated in mediating neuroprotection after hypoxic preconditioning [31,32]. BDNF, SDF-1α and VEGF can affect both neuronal and vascular function in the ischemic brain. BDNF was originally discovered through its neuronal effects and then later found to also have vascular effects, whereas SDF-1α and VEGF were originally discovered through their angiogenic effects and then later found to also have neuroprotective activity [33]. BDNF is a potent growth factor involved in recovery following cerebral ischemia [15,16]. Altered BDNF plasma levels or association of BDNF genotypes indicate that this growth factor may be involved in the physiological response to stroke in humans [15]. SDF-1α, a CXC chemokine produced by bone marrow stromal cells, a potent chemo-attractant for hematopoietic stem cells, is constitutively expressed by all tissues [34]. Stumm et al. demonstrated that focal cerebral ischemia causes an increase in endothelial SDF-1α expression in regions adjacent to the infarcted area [35]. Recently, intracerebral administration of SDF-1α was reported to induce neuroprotection against neurotoxic insult, and induce increased bone marrow-derived cell targeting of the ischemic brain, thereby reducing the volume of cerebral infraction and improving neural plasticity [17]. VEGF is an angiogenic peptide that also exerts a large number of diverse neuronal effects in the central nervous system [36]. For these reasons, BDNF, SDF-1α and VEGF are potent candidates in ischemic preconditioning; however, little is known about the involvement of them in EA preconditioning in ischemic brains. Therefore, we examined whether BDNF, SDF-1α and VEGF were involved in neuroprotective effects of EA preconditioning in focal cerebral ischemia. We found that EA preconditioning increased the brain tissue content of BDNF and upregulated the production of SDF-1α in the plasma. In addition, a large number of BDNF- and SDF-1α-positive cells in the ischemic cortex were observed in the EA group. Moreover, the present immunofluorescence data showed that BDNF immunoreactivities were colocalized with NeuN immunoreactivities and SDF-1α immunoreactivities with CD31 immunoreactivities, indicating that the effect of EA pretreatment on BDNF expression was neuron-specific and SDF-1α was vessel-specific in the ischemic cortex. These current results strongly suggest that upregulation of neuronal BDNF and vascular SDF-1α after EA pretreatment might be an important protective mechanism of tolerance against ischemia. Interestingly, SDF-1α production was upregulated in a time-dependent manner following EA stimulation, while VEGF remained unchanged after EA pretreatment in the plasma. A few studies have shown that VEGF production was increased by EA [37]. These different results might be due to differences in the severity of cerebral ischemia or the use of different acupoints in this and previous studies.

## Conclusions

The present study showed that EA stimulation at the GV20 and GV14 acupoints before focal cerebral ischemia has neuroprotective potential mediated, at least in part, by increased BDNF and SDF-1α expression. The present findings suggest a novel mechanism of EA pretreatment-induced tolerance against cerebral ischemic injury in mice.

## Abbreviations

EA: Electroacupuncture; BDNF: Brain-derived neurotrophic factor; SDF-1α: Stromal cell derived factor-1α.

## Competing interests

The authors declare that there are no financial competing interests.

## Authors’ contributions

Conception and design: HKS and BTC. Acquisition of data: JHK, KHC, YJJ and HNK. Analysis and interpretation of data: SSB, BTC and HKS. Drafting the manuscript: BTC and HKS. All authors read and approved the final manuscript.

## Pre-publication history

The pre-publication history for this paper can be accessed here:

http://www.biomedcentral.com/1472-6882/13/22/prepub
